# Plant and Animal Protein Intake and Transitions From Multimorbidity to Frailty and Mortality in Older Adults

**DOI:** 10.1002/jcsm.13729

**Published:** 2025-02-19

**Authors:** Aitana Vázquez‐Fernández, Francisco F. Caballero, Humberto Yévenes‐Briones, Ellen A. Struijk, Ana Baylin, Teresa T. Fung, Esther Lopez‐Garcia

**Affiliations:** ^1^ Department of Preventive Medicine and Public Health, School of Medicine Universidad Autónoma de Madrid Madrid Spain; ^2^ Department of Nutritional Sciences, School of Public Health University of Michigan Ann Arbor Michigan USA; ^3^ Department of Nutrition Simmons University Boston Massachusetts USA; ^4^ Department of Nutrition Harvard T. H. Chan School of Public Health Boston Massachusetts USA

**Keywords:** frailty, multimorbidity, older adults, plant and animal proteins, transitions

## Abstract

**Background:**

Multimorbidity is the most common chronic condition experienced among older adults. It is unknown which amount and source of protein influences the development of frailty and mortality in patients with multimorbidity. We aimed to examine the association of plant and animal sources of protein intake with frailty and mortality among this type of patients.

**Methods:**

This longitudinal study included 1868 participants aged ≥ 60 years from the Seniors‐ENRICA cohort in Spain with multimorbidity, defined as having 2 or more clinician‐diagnosed chronic diseases. Habitual diet was assessed at baseline (2008–2010) with a validated computerized diet history. Participants underwent repeated physical examinations (in 2013, 2015 and 2017) for assessment of frailty (≥ 3 criteria from the frailty phenotype: low physical activity, slow walking speed, muscle weakness, weight loss and exhaustion). All‐cause mortality was assessed up to January 2022. Analyses were conducted using Cox proportional hazard models and multistate models adjusted for sociodemographic, lifestyle and other dietary factors.

**Results:**

Mean consumption of protein was 90.2 (standard deviation [SD]: 26.8) g/day, which represents 18.7% of the total energy intake and 1.23 (0.39) g per kg of body weight per day. Plant protein represented 6.16% of the energy intake, while animal protein represented 12.5%. During a median follow‐up of 12.9 (range: 11.7–13.9) years, we documented 196 incident cases of frailty and 490 deaths; of these mortality cases, 83 individuals died after a frailty diagnosis. Higher intake of total protein was associated with decreased risk of frailty (hazard ratio [HR] for tertile 3 vs. tertile 1: 0.66; 95% confidence interval [CI]: 0.45, 0.96; *p* trend: 0.03). In multistate models, higher fish protein intake decreased the risk in the progression from multimorbidity to frailty (HR per 1‐SD increment: 0.81 [95% CI: 0.68, 0.97]), and higher plant protein decreased the risk of progressing from multimorbidity to mortality (0.86 [0.75, 0.98]). In the progression from frailty to mortality, estimates for total, plant and animal protein showed increased risk (HR for 1 SD increment in total protein: 1.38 [1.05, 1.81]; HR for plant protein: 1.29 [1.01, 1.67]; HR for animal protein: 1.41 [1.04, 1.92]). No significant associations were found between meat protein and dairy protein in any transition.

**Conclusions:**

In individuals with multimorbidity, higher protein intake, especially fish protein, was associated with lower risk of subsequent frailty, whereas plant protein intake was associated with lower risk of mortality. Higher total protein intake, however, might be detrimental in patients with multimorbidity and frailty.

**Trial Registration:**

ClinicalTrials.gov identifier: NCT02804672.

## 1 Introduction

Multimorbidity is defined as the coexistence of multiple chronic conditions in an individual and is associated with an increased risk of adverse health outcomes including reduced functional status, frailty, disability, lower quality of life and increased mortality [1, 2]. People affected by multimorbidity often experience fragmentation of care, greater and inadequate use of health services and polypharmacy, which may increase the risk of low adherence and adverse drug reactions. Handling patients with multimorbidity represents a daily challenge for physicians and health systems [3]. Multimorbidity is closely related to the ageing process of the population, becoming the most common chronic condition experienced in adults.

In parallel, the concept of frailty has gained considerable attention. Frailty is characterized by a decline in an individual's physiological state and functional capacity and is often manifested by loss of strength, endurance, mobility and overall adaptive capacity [4]. Frail individuals are at increased risk for adverse health events [5]. Although frailty and multimorbidity are distinct concepts, they often overlap in clinical practice [6]. Most frail individuals present multimorbidity, but a smaller fraction of individuals with multimorbidity are frail; the risk factors why some individuals progress from multimorbidity to frailty are unknown [7]. Thus, it is unclear whether frailty might be a mediator between multimorbidity and mortality.

Previous research has linked overall diet quality to lower risk of frailty and mortality [8, 9]. Protein has received particular attention since older adults may need higher intake because of their decreased capacity to metabolize this nutrient and the additional protein requirements in those with chronic diseases and malnutrition [10]. However, high protein intake may also be detrimental to patients with altered renal function, and protein restriction is a common recommendation in clinical practice for them [11].

While higher total protein intake has been associated with lower risk of frailty [12], the evidence is conflicting about which source of protein has a greater protective effect. Results from population studies favour the intake of vegetable proteins [13, 14, 15], while interventions in older adults favour supplementation with animal protein [16, 17]. In addition, new dietary guidelines are shifting towards plant‐based diets partially based on the results from cohort studies showing a beneficial effect of plant versus animal protein on total mortality [18, 19, 20, 21], but it is still unclear how these recommendations could impact the older population. Therefore, the aim of this study was to assess the association between total and different sources of protein intake and risk of frailty and mortality among community‐dwelling older adults with multimorbidity.

## 2 Methods

### 2.1 Study Design and Participants

Baseline data were obtained from the ENRICA cohort study, established between 2008 and 2010 among a representative sample of community‐dwelling individuals in Spain (*n* = 12 948). Study participants aged 60 years and older comprised the Seniors‐ENRICA cohort (*n* = 3289) [S1], from which information for this study was collected. Information on socio‐demographic variables, health status, lifestyle and morbidity was collected at baseline by a computer‐assisted telephone interview. Then, two subsequent home visits were conducted to obtain biological samples, perform a diet history and a physical examination. Further waves of data collection were performed in 2013, 2015 and 2017 following the same procedure. The study was conducted following the Declaration of Helsinki guidelines and was approved by the Clinical Research Ethics Committee of ‘La Paz’ University Hospital in Madrid, Spain. Written informed consent was obtained from all study participants.

### 2.2 Study Variables

#### 2.2.1 Diet

Information on food consumption at baseline was obtained by trained interviewers through a validated computerized diet history developed from that used in the EPIC‐Spain cohort study [S2]. Study participants reported their weekly consumption of 880 foods classified according to their nutrient content. Nutrient intakes were estimated using standard Spanish food consumption tables supplemented with international tables [S3, S4, S5]. The validity of the diet history has been previously evaluated by comparing its results with seven 24‐h recalls over a 1‐year period in a subsample of participants [S2]. Energy‐adjusted Pearson's correlation coefficients ranged from 0.27 to 0.71 across food groups and nutrients [S2]. Specifically, the correlation for protein intake was 0.50. Regarding the reliability of the diet history, the intraclass correlation coefficient between two diet assessments was > 0.40 for most foods and nutrients. The percentage of energy from each macronutrient was used instead of absolute intake to reduce bias due to underreporting of food consumption and to reflect dietary composition. The energy percentage of protein intake was classified into total protein, plant protein and animal protein. Animal protein was further divided into meat, dairy, fish and egg protein.

#### 2.2.2 Multimorbidity Assessment

Morbidity information was collected at baseline through self‐reported, dichotomous questions asked during the computer‐assisted telephone interview about the presence or absence of a list of 21 available clinician‐diagnosed chronic diseases. This list included asthma or chronic bronchitis, heart attack or infarction, thrombosis or stroke, heart failure, sleep apnoea, osteoarthritis or rheumatism, rheumatoid arthritis, hip fracture, gallstones, intestinal polyps, stomach or duodenal ulcer, liver cirrhosis, cataracts, depression, Parkinson's disease, dementia, Alzheimer's disease, cancer, hypertension, diabetes or hypercholesterolemia. The presence of two or more conditions from this list was classified as a case of multimorbidity.

#### 2.2.3 Frailty Assessment

At baseline, since objective measurements of grip strength and gait speed were not available, prevalent cases of frailty were identified as those with at least three of the frailty components from the Fatigue, Resistance, Ambulation, Illnesses and Loss of Weight (FRAIL) scale, which is based on self‐reported information [S6]. Then, information on frailty status during follow‐up was collected in 2013, 2015 and 2017 during the home visit physical examination, with an exhaustive battery of tests to assess physical performance, including grip strength and gait speed. With this information, incident frailty was assessed using the frailty phenotype proposed by Fried et al. [5], which consists of the presence of at least three of the following criteria: (a) unintentional weight loss of ≥ 4.5 kg in the preceding year; (b) exhaustion, based on an affirmative response to any of the following questions from the Center for Epidemiologic Studies Depression Scale: ‘I felt that anything I did was a big effort’ or ‘I felt that I could not get going’ at least 3 or 4 days a week; (c) low physical activity, defined as walking ≤ 2.5 h/week for men and ≤ 2.0 h/week for women; (d) slow walking speed, defined as the lowest cohort‐specific quintile of gait speed over 2.44 m, adjusted for sex and height; and (e) muscle weakness, set as the cohort‐specific lowest quintile of grip strength, measured with a Jamar dynamometer in the dominant hand, adjusted for sex and body mass index (BMI). These criteria were kept constant during all the follow‐up assessments. If responses on ≥ 3 criteria were missing, we assumed as missing on frailty status and the person‐time for that period was excluded. However, if data were missing only for 1 or 2 criteria, we were able to assess frailty status using the remaining frailty components and assuming the missing components to be absent.

#### 2.2.4 Mortality

Vital status was ascertained by computerized search of the Spanish National Death Index, which contains information on the vital status of all residents of Spain from baseline to the most recent update of mortality information on 30 January 2022 [S7].

#### 2.2.5 Potential Confounders

Sociodemographic data collected at baseline included sex, age and level of education (primary, secondary or university). Habitat was approached by size (< 10 000; 10 000–100 000; > 100 000–500 000; > 500 000 inhabitants). Tobacco use was categorized as never, former and current smoker. Alcohol consumption was considered as grams of ethanol intake per day. Physical activity was assessed using the EPIC cohort questionnaire validated in Spain and expressed in metabolic equivalent task hours/week (METs‐h/week) [S8]. Sedentary behaviour was considered as TV hours/day and sleep time as sleep hours/day reported, respectively. Participants also reported the number of drugs used. Total energy intake was derived from information of the diet history and expressed in kilocalories per day. The Mediterranean Diet Adherence Screener (MEDAS) was used to assess adherence to the Mediterranean diet pattern, as an indicator of overall diet quality; this score ranged from 0 to 14, with higher values indicating greater adherence [S9]. Weight and height were measured under standardized conditions, and BMI was calculated as weight (kg) divided by the square of height (m^2^) and classified as < 25, 25–29.9 and ≥ 30 kg/m^2^. Glomerular filtration rate (eGFR) was approached using serum creatinine and the simplified 4‐variable Modification of Diet in Renal Disease (MDRD) Study equation [S10].

### 2.3 Statistical Analyses

From the initial sample of 3289 participants at baseline, we excluded 68 individuals with missing information on multimorbidity, 203 with missing information on frailty status at baseline and 89 with prevalent frailty. In addition, 13 participants with implausible or missing energy intake were excluded. Of the 2916 remaining eligible participants, only those with multimorbidity (defined as two or more diseases) were selected; thus, the analytical sample comprised 1868 individuals (Figure S1).

Differences in sociodemographic, lifestyle and clinical variables across tertiles of each protein source were assessed with the chi‐square test for the qualitative variables and the ANOVA test for the continuous variables. We used cause‐specific hazards models to calculate relative risks, estimated by hazard ratios (HR), and their 95% confidence interval (CI) to assess the association between intakes of total, plant and animal (including meat, dairy, egg and fish) protein, categorized into tertiles (with the lowest one as reference), and frailty status or all‐cause mortality, respectively. Person‐years were calculated from baseline in 2008–2010 until the occurrence of the outcome (frailty or death), loss of follow‐up or end of the study (30 January 2022), whichever came first. Two models were fitted with sequential adjustment for potential confounders. The first model was adjusted for age, sex and number of chronic diseases at baseline. The second model was additionally adjusted for educational level, habitat, smoking status, alcohol consumption, physical activity, hours of TV watching, sleep duration, number of drugs, BMI, energy intake, percentage of energy from saturated, monounsaturated and polyunsaturated fats and the MEDAS score. All models included mutual adjustment for the percentage of energy derived from the alternative sources of protein (e.g., plant protein was adjusted for animal protein; dairy protein was adjusted for plant protein and all other sources of animal protein, etc.). In addition, tests for linear trends were conducted by modelling the median protein intake of each tertile as a continuous variable.

Further analyses were performed with multistate models (three possible states) using the Weibull distribution to estimate the impact of 1‐SD increment in each type of protein intake on the transitions from (1) multimorbidity to frailty status, (2) multimorbidity to death and (3) frailty status to death (Figure S2). Multistate models depict how an individual moves through various states under the assumption that each successive state depends only on the current state [S11, S12]. The three transitions could occur at any time during follow‐up, but once an individual transitioned to a state, he/she was considered to remain in that state until the transition to death or the end of follow‐up, as we considered that frailty in our participants could not be reversed when they met ≥ 3 criteria of frailty phenotype. These models provide a transition structure that allows understanding the progression of adverse health conditions.

Lastly, additional multistate models were formulated adjusting for eGFR as a continuous variable to account for the effect of renal function and adjusting for dietary vitamin D, since a low intake might be associated with a greater risk of frailty. Statistical tests were 2‐sided with a *p* value < 0.05 and performed using Stata software (version 17.0; Stata Corp., College Station).

## 3 Results

Mean age of the participants with multimorbidity was 69.7 (SD 6.62) years. The average number of chronic conditions was 3.10 (1.28), with 40.9% participants having 2 diseases, 28.3% having 3 diseases and 30.8% having 4 diseases or more. Baseline characteristics of the study participants across tertiles of different sources of protein intake are shown in Table 1. For total protein intake, individuals in the highest tertile were more often women, in comparison with those in the lowest tertile. Individuals in the highest tertile of plant proteins were less likely to have secondary or university studies; also, they were less frequently current smokers. For animal protein, those with higher intake showed an increased BMI compared to those with lower intake. In addition, participants in the highest tertiles of total, plant or animal protein intake reported lower alcohol and total energy intake and showed better adherence to the MEDAS score than those in the lowest tertiles.

**TABLE 1:** Characteristics of participants in the study (*N* = 1868).

	Total protein, % energy		Plant protein, % energy		Animal protein, % energy	
	Tertile 1	Tertile 3	Tertile 1	Tertile 3	Tertile 1	Tertile 3
Participants, *n*	623	622	623	622	623	622
Sex, women %	54.7	64.3**	55.7	63.0*	57.9	62.5
Age, years	69.4 (6.36)	69.7 (6.34)	69.2 (6.54)	70.0 (6.58)	69.8 (6.41)	69.5 (6.46)
Educational level, (secondary/university studies), %	39.3	37.1	42.6	33.4**	36.8	38.4
Habitat (> 100 000 inhabitants), %	44.1	49.7	47.8	47.8	45.6	51.0
Tobacco consumption (current smoker), %	13.8	9.0*	12.7	7.9*	12.7	9.5
Physical activity, METs‐h/week	20.2 (14.3)	21.4 (15.2)	20.3 (14.0)	20.5 (15.0)	20.3 (14.7)	21.4 (15.2)
TV watching, h/week	19.3 (11.6)	19.4 (11.5)	19.3 (11.9)	18.7 (10.7)	19.1 (11.7)	19.5 (11.8)
Sleeping time, h/week	6.85 (1.42)	6.81 (1.45)	6.85 (1.47)	6.78 (1.42)	6.83 (1.45)	6.82 (1.45)
BMI, kg/m^2^	28.7 (3.92)	29.4 (4.65)***	29.4 (4.50)	28.9 (4.37)***	28.5 (4.01)	29.5 (4.71)***
Chronic diseases at baseline, number	3.04 (1.38)	3.15 (1.24)**	3.04 (1.26)	3.19 (1.27)	3.09 (1.27)	3.09 (1.21)*
Drug use, number	1.17 (0.95)	1.19 (0.98)	1.13 (0.93)	1.17 (1.00)	1.16 (0.96)	1.18 (0.95)
Alcohol consumption, g/day	14.4 (21.8)	4.8 (10.2)***	14.8 (22.5)	4.9 (11.1)***	11.4 (19.2)	6.4 (12.7)***
Energy intake, kcal/day	2187 (597)	1747 (525)*	2030 (605)	1863 (539)**	2133 (586)	1779 (527)**
MEDAS, points	6.58 (2.12)	7.40 (1.52)***	6.74 (1.69)	7.55 (1.75)***	6.72 (2.21)	7.28 (1.55)***
eGFR, mL/min/1.73 m^2^	79.8 (17.8)	78.06 (15.7)	78.1 (15.3)	78.8 (17.3)	79.5 (17.2)	77.5 (15.6)

*Note*: Continuous variables are given as means (SD). *p* values based on chi‐square test for qualitative variables or ANOVA test for continuous variables.

Abbreviations: BMI: body mass index; eGFR: estimated glomerular filtration rate; MEDAS: Mediterranean Diet Adherence Screener; METs: metabolic equivalents tasks.

Statistically significant (*p < 0.05, **p < 0.01, ***p < 0.001).

Table 2 shows the distribution of macronutrients across tertiles of protein intake. Mean consumption of total protein was 90.2 (26.8) g/day, which represents 18.7% of the total energy intake and a mean of 1.23 (0.39) g/kg per day. Individuals in the lowest tertile of total protein intake had a mean consumption of 81.9 (22.6) g/day while those in the highest tertile had a mean intake of 98.6 (30.8) g/day. Plant protein represented 6.16% of the total energy intake, while animal protein represented 12.5%. In terms of animal protein, the most consumed source was meat protein, followed by dairy, fish and egg protein. Energy from fats represented 35.7% of the total intake, and energy from carbohydrates was 42.5%.

**TABLE 2:** Distribution of total, plant and animal protein and macronutrients across tertiles of protein intake.

	Protein intake, % energy			
	Overall (*N* = 1868)	Tertile 1 (*n* = 623)	Tertile 2 (*n* = 623)	Tertile 3 (*n* = 622)
Total protein, % energy	18.7 (3.70)	15.0 (1.34)	18.3 (0.85)	22.8 (2.89)
Total protein, g/day	90.2 (26.8)	81.9 (22.6)	90.2 (23.7)	98.6 (30.8)
Total protein, g/kg/day	1.23 (0.39)	1.11 (0.30)	1.24 (0.35)	1.35 (0.48)
Plant protein, % energy	6.16 (1.40)	4.71 (1.91)	6.10 (0.30)	7.67 (0.94)
Plant protein, g/day	30.0 (10.3)	23.9 (8.05)	30.6 (8.74)	35.5 (10.5)
Animal protein, % energy	12.5 (3.96)	8.68 (1.34)	12.0 (0.88)	16.9 (3.16)
Animal protein, g/day	60.2 (22.7)	38.8 (7.87)	57.4 (4.81)	84.4 (20.4)
Meat protein, g/day	25.4 (15.5)	14.3 (6.88)	24.0 (8.94)	37.9 (17.8)
Dairy protein, g/day	16.0 (10.3)	11.5 (6.12)	15.4 (7.59)	21.2 (13.2)
Fish protein, g/day	14.7 (12.4)	9.76 (5.71)	13.8 (7.10)	20.5 (17.9)
Egg protein, g/day	2.97 (2.80)	2.32 (2.23)	2.92 (2.47)	3.68 (3.40)
Total fat, % energy	35.7 (6.60)	36.3 (6.55)	35.1 (6.80)	35.7 (6.57)
Saturated fat, % energy	10.7 (3.18)	10.9 (2.85)	10.5 (3.59)	10.7 (3.18)
Monounsaturated fat, % energy	15.6 (3.70)	15.9 (3.86)	15.6 (3.57)	15.3 (3.66)
Polyunsaturated fat, % energy	6.10 (2.25)	6.28 (2.32)	6.17 (2.30)	5.83 (2.11)
Total cholesterol, mg/day	298 (133)	296 (142)	294 (123)	305 (134)
Carbohydrates, % energy	42.5 (7.36)	44.2 (7.17)	43.0 (6.94)	40.2 (7.42)

*Note:* All values are means and standard deviations. Energy from alcohol consumption is not shown in the table above.

During a median follow‐up of 12.9 (range: 11.7–13.9) years, 196 cases of incident frailty and 490 deaths were identified. Of these mortality cases, 83 individuals were first diagnosed as frail. Regarding incident frailty, participants with higher total protein intake, compared with those with the lowest intake, had a reduced risk of frailty in the fully adjusted model (HR tertile 3 vs. tertile 1: 0.66 [95% CI: 0.45, 0.96]; *p*‐trend: 0.03) as given in Table 3. When plant protein was assessed separately, no association was found. However, for animal protein, individuals in the second tertile had a significantly lower risk of incident frailty compared to those in the first tertile in the fully adjusted model, although the linear trend was not significant. Also, individuals in the highest tertiles of fish intake, compared with those in the first tertile, showed a lower risk of incident frailty in the fully adjusted model (HR tertile 3. vs. tertile 1: 0.67 [0.45, 0.99]; *p*‐trend: 0.05). No significant associations were found for meat and egg protein and frailty.

**TABLE 3:** Association between tertiles of protein intake and frailty in individuals with multimorbidity (*N* = 1868).

	Protein intake, % energy			
	Tertile 1	Tertile 2	Tertile 3	*p* for trend
Participants, *n*	623	623	622	
Total protein				
Person‐years/*n* cases	4068/74	3930/62	3891/60	
Model 1	1.0	0.81 (0.58, 1.13)	0.75 (0.53, 1.05)	0.10
Model 2	1.0	0.77 (0.54, 1.10)	**0.66 (0.45, 0.96)**	**0.03**
Plant protein				
Person‐years/*n* cases	4032/63	3942/67	3916/66	
Model 1	1.0	1.02 (0.72, 1.45)	0.90 (0.63, 1.29)	0.55
Model 2	1.0	1.03 (0.71, 1.49)	0.96 (0.62, 1.48)	0.85
Animal protein				
Person‐years/*n* cases	3937/74	4041/56	3912/66	
Model 1	1.0	0.75 (0.53, 1.07)	0.88 (0.62, 1.24)	0.51
Model 2	1.0	**0.68 (0.47, 0.99)**	0.80 (0.55, 1.18)	0.32
Meat protein				
Person‐years/*n* cases	3835/76	4013/55	4042/65	
Model 1	1.0	0.74 (0.52, 1.05)	0.95 (0.67, 1.33)	0.84
Model 2	1.0	0.79 (0.55, 1.14)	0.88 (0.61, 1.25)	0.53
Dairy protein				
Person‐years/*n* cases	4013/67	4105/47	3771/82	
Model 1	1.0	**0.60 (0.41, 0.88)**	1.02 (0.73, 1.43)	0.55
Model 2	1.0	**0.60 (0.40, 0.88)**	1.01 (0.69, 1.48)	0.69
Fish protein				
Person‐years/*n* cases	3817/81	4113/61	3959/54	
Model 1	1.0	0.72 (0.51, 1.00)	**0.66 (0.47, 0.94)**	**0.02**
Model 2	1.0	0.70 (0.49, 1.02)	**0.67 (0.45, 0.99)**	**0.05**
Egg protein				
Person‐years/*n* cases	3917/65	4032/64	3941/67	
Model 1	1.0	1.05 (0.74, 1.50)	1.12 (0.79, 1.58)	0.52
Model 2	1.0	1.09 (0.76, 1.57)	1.16 (0.81, 1.66)	0.43

*Note:* Estimates are hazard ratios (95% confidence interval). Model 1: Cox regression model adjusted for age, sex and number of chronic diseases at baseline. Model 2: additionally adjusted for educational level (≤ primary, secondary or university), habitat (< 10 000; 10 000–100 000; > 100 000–500 000; > 500 000), smoking status (never, former, current smoker), alcohol consumption (tertiles of g/day), physical activity (tertiles of METs‐h/week), hours of TV (tertiles of h/day), sleeping time (tertiles of h/day), number of drugs used, BMI (< 25, 25–29.9, ≥ 30 kg/m^2^), energy intake (tertiles of kcal/day), % of saturated fat, % of monounsaturated fat, % of polyunsaturated fat and diet quality (MEDAS score, tertiles). All models include mutual adjustment for percentages of energy derived from each other source of protein (e.g., vegetable protein was adjusted for animal protein; dairy protein was adjusted for plant protein and all other types of animal protein, etc.). Results that are statistically significant are highlighted.

Abbreviations: BMI: body mass index; MEDAS: Mediterranean Diet Adherence Screener; MET: metabolic equivalent tasks.

The association between protein intake and all‐cause mortality is shown in Table S1. No association was observed for total protein intake and plant or animal protein and risk of death.

In Table 4, total protein was associated with decreased risk of progressing from multimorbidity to frailty (HR per 1‐SD increment: 0.82 [95% CI: 0.69, 0.97]); this association was due to fish protein (0.81 [0.68, 0.97]). On the other hand, an increment in plant protein was associated with decreased risk of progressing from multimorbidity to mortality (0.86 [0.75, 0.98]). However, in the progression from frailty to mortality, estimates for increments of total, plant and animal protein showed increased risk (HR for total protein: 1.38 [1.05, 1.81]; HR for plant protein: 1.29 [1.01, 1.67]; HR for animal protein: 1.41 [1.04, 1.92]). No significant associations were found between meat, dairy and egg protein in any transition. Renal function assessed through the eGFR did not modify the results found in the multistate models, neither did adjustment for dietary vitamin D, as shown in Tables S2 and S3.

**TABLE 4:** Progressions from multimorbidity to frailty and mortality per 1‐SD increment of protein intake.

Protein intake, % energy								
	*N*/*n*	Total protein	Plant protein	Animal protein	Meat protein	Dairy protein	Fish protein	Egg protein
1‐SD increment, % energy		5.06	4.40	3.17	1.78	1.63	1.39	1.03
Multimorbidity to frailty	1868/196	**0.82 (0.69, 0.97)**	1.02 (0.84, 1.23)	**0.81 (0.68, 0.97)**	0.96 (0.82, 1.12)	0.89 (0.75, 1.05)	**0.81 (0.68, 0.97)**	1.00 (0.87, 1.16)
Multimorbidity to mortality	1868/407	0.97 (0.86, 1.08)	**0.86 (0.75, 0.98)**	0.95 (0.85, 1.07)	0.95 (0.85, 1.05)	0.99 (0.88, 1.11)	0.99 (0.89, 1.09)	1.03 (0.93, 1.13)
Frailty to mortality	196/83	**1.38 (1.05, 1.81)**	**1.29 (1.01, 1.67)**	**1.41 (1.04, 1.92)**	1.27 (0.94, 1.70)	1.24 (0.92, 1.67)	1.06 (0.76, 1.48)	1.23 (0.91, 1.67)

*Note:* Multistate models adjusted for age, sex, number of chronic diseases at baseline, educational level (≤ primary, secondary or university), habitat (< 10 000; 10 000–100 000; > 100 000–500 000; > 500 000), smoking status (never, former, current smoker), alcohol consumption (tertiles of g/day), physical activity (tertiles of METs‐h/week), hours of TV (tertiles of h/day), sleeping time (tertiles of h/day), number of drugs used, BMI (< 25, 25–29.9, ≥ 30 kg/m^2^), energy intake (tertiles of kcal/day), % of saturated fat, % of monounsaturated fat, % of polyunsaturated fat and diet quality (MEDAS score, tertiles). All models include mutual adjustment for percentages of energy derived from each other type of protein (e.g., vegetable protein was adjusted for animal protein; dairy protein was adjusted for plant protein and all other types of animal protein, etc.). Estimates are hazard ratios (95% confidence interval). Results that are statistically significant are highlighted.

Abbreviations: METs: metabolic equivalent tasks; BMI: body mass index; MEDAS: Mediterranean Diet Adherence Screener.

## 4 Discussion

In this prospective study, we examined the progression from multimorbidity to frailty, from multimorbidity to mortality and from frailty to mortality. Participants with a higher total protein intake, particularly fish protein, had a lower risk of frailty, independently of sociodemographic, lifestyle and overall diet quality. Plant protein intake was associated with lower risk of mortality. We also found that once frailty is established in patients with multimorbidity, higher protein intake was associated with increased risk of mortality.

A growing body of evidence suggests that higher total protein intake is beneficial and may reduce and delay the onset of frailty [14, 22, 23, 24]. According to our results, in individuals with established multimorbidity, this is the case even when overall diet quality and other macronutrients such as fat intake are considered, which implies that protein intake by itself influences frailty independently of other dietary components. This is important because overall diet quality has also been associated with a lower risk of frailty [8]. In this line, the *Study Group on meeting protein needs of older people* (PROT‐AGE) group recommends 1.0–1.2 g/kg body weight per day for this subgroup of the population [10]. In our study, even those with lower total protein intake were found to be above the recommendations provided in the latest report by the Spanish Agency for Food Security and Nutrition (AESAN), which recommended 0.83 g/kg body weight per day for this age group [25]. Our results suggest that higher intakes may still be beneficial for preventing frailty.

The potential mechanisms through which increased protein intake may prevent frailty are diverse [26]. Frailty is associated with malnutrition, with frail individuals frequently lacking essential nutrients, including protein. Malnutrition contributes to all the criteria outlined in Fried's frailty definition. Notably, higher protein intake has been linked to improvements in weight loss, walking speed [27], grip strength [28] and muscle mass [29]. With ageing, individuals tend to lose muscle mass and muscle strength in a process known as sarcopenia [30]. This loss can be attributed to a diminished muscle protein anabolic response, which may stem from insufficient nutrition or an impaired response to nutrients.

On the other hand, in patients with multimorbidity and frailty, higher protein was associated with increased mortality. A plausible hypothesis for this result is that these patients may have many altered systems, including kidney function, and high‐protein diets cause dilation of glomerular afferent arterioles, which lead to glomerular hyperfiltration and progression of chronic kidney disease [11]; however, adjusting the models for the estimated glomerular filtration rate did not change the results, suggesting that renal function does not explain these findings in our study.

As new dietary guidelines lean towards plant‐based diets because of their impact on the environment and mortality, it is imperative to clarify what recommendations should be given to older adults who are at higher risk of becoming frail. Not only do they have higher protein requirements, but their muscle synthesis processes are not as efficient [31]. A previous study suggested that to meet the overall nutrient recommendations, at least half of the protein intake would have to be from animal protein, with variations depending on gender and age [32]. In addition, one of the main hypotheses for a different effect of plant and animal protein is its amino acid composition. Animal protein is believed to have a higher biological value amino acid profile that promotes muscle synthesis processes, often referred to as high quality protein. Of all the amino acids, leucine, a branched‐chain amino acid found primarily in animal‐source protein, plays a key role in the signalling pathways of muscle protein synthesis. In a prospective study conducted in the same cohort as in our study, a higher intake of dietary leucine, mainly from animal sources, was associated with a lower risk of frailty [33].

When analysing protein sources, we found that animal protein was associated with lower incident frailty, which is consistent with other studies [22, 34]. Nevertheless, there are also some cross‐sectional studies reporting that the lower risk of frailty is independent of the source of protein consumed, animal or plant protein [35, 36], suggesting that the type of protein is not so important if an adequate level of intake is achieved. A recent study conducted in a large cohort, with information on cumulative intake over 22 years of follow‐up, found that a higher intake of plant protein was associated with a lower risk of frailty while this result was not observed for animal protein [14]. Nonetheless, when looking at protein intake in the short term, they also found an inverse association between animal protein and frailty. The authors argued that as the ageing process progresses and chronic diseases develop, animal protein may become a more important contributor to frailty. Given that the individuals in our study already had multimorbidity, that is, they had already developed these chronic diseases, this may be one of the reasons why plant protein was not associated with incident frailty in our case.

Among animal protein sources, fish protein showed the greatest protective effect in our population, even after adjusting for fat intake, which is one of the nutrients responsible for the benefits of fish. A cross‐sectional study found that higher intake of oily fish was associated with lower frailty scores, although this effect cannot be attributed to fish protein alone [37]. Another prospective study found that replacing meat protein with fish protein reduced the risk of frailty [38].

The strengths of this study are its prospective design and the length of the follow‐up. The use of multistate models allowed us to observe transitions between states considering the entire study population, even in those transitions where the number of individuals was smaller. We also used an objective and well‐recognized frailty assessment tool such as the frailty phenotype.

This study also has some limitations. Although we used a validated dietary history with a good correlation with protein intake, some margin of error is expected since information was self‐reported and only collected at baseline; however, the protein intake measured was similar or slightly higher than in other studies in Spain, which used other instruments to assess habitual diet [39, 40]. In addition, changes in protein intake during follow‐up were not considered. Our analysis focused on the impact of plant and animal protein; however, we did not consider protein quality, in terms of amino acid composition and digestibility. Therefore, the results need to be put in the context of prioritizing diets with protein adequacy, of particular importance for older adults, with increased nutrient requirements.

On the other hand, multimorbidity was defined based on self‐reported clinician diagnoses, while the use of electronic health records is preferred. Furthermore, despite adjustment for sociodemographic, lifestyle and dietary factors, some residual confounding may persist as with any observational study. Although we adjusted our results for the number of chronic conditions at baseline, we did not have information on the date of diagnosis of those conditions; therefore, the possibility of reverse causality cannot be excluded, since individuals with multimorbidity may have experienced metabolic alterations that affected their dietary habits, including protein intake, which may ultimately have altered their risk of frailty and mortality. Despite dietary adjustments, we cannot exclude the possibility that other nutrients from animal and plant sources may be partially responsible for the associations found. Finally, generalization of these results should be made with caution since characteristics of the population considered, like their habitual diet or social determinants of health, as well as the healthcare system of Spain, may have contributed to the results.

In conclusion, among individuals with multimorbidity, higher protein intake, especially fish protein, was associated with lower risk of frailty, whereas plant protein was associated with lower risk of mortality, and higher protein, independently of the source, was found to be detrimental in patients with multimorbidity and frailty. The clinical implication of these results, in particular, the finding that higher protein was associated with increased mortality, requires additional studies that can inform on the causality of the relation. Future research should aim to better characterize optimal dietary exposures for patients with multimorbidity, including more specific food types, and to guide more targeted recommendations for healthier ageing.

## Ethics Statement

The study was conducted according to the Declaration of Helsinki guidelines and was approved by the Clinical Research Ethics Committee of ‘La Paz’ University Hospital in Madrid, Spain. Written informed consent was obtained from all study participants.

## Conflicts of Interest

The authors declare no conflicts of interest.

## Supporting information


**Table S1** Association between tertiles of protein intake and mortality in individuals with multimorbidity (*N* = 1868).
**Table S2** Progressions from multimorbidity to frailty and mortality per 1‐SD increment of protein intake, with additional adjustment for estimated glomerular filtration rate.
**Table S3** Progressions from multimorbidity to frailty and mortality per 1‐SD increment of protein intake, with additional adjustment for dietary vitamin D.
**Figure S1** Participants’ flow chart.
**Figure S2** Progressions from multimorbidity to frailty/mortality.
