# Supplementary material for: Plant and Animal Protein Intake and Transitions From Multimorbidity to Frailty and Mortality in Older Adults
Source: J Cachexia Sarcopenia Muscle. 2025 Feb 19;16(1):e13729. doi: 10.1002/jcsm.13729 (PMC11839239; doi:10.1002/jcsm.13729)
Supplement: Supplementary file 1 — Table S1 Association between tertiles of protein intake and mortality in individuals with multimorbidity (N = 1868). Table S2 Progressions from multimorbidity to frailty and mortality per 1‐SD increment of protein intake, with additional adjustment for estimated glomerular filtration rate. Table S3 Progressions from multimorbidity to frailty and mortality per 1‐SD increment of protein intake, with additional adjustment for dietary vitamin D. Figure S1 Participants’ flow chart. Figure S2 Progressions from multimorbidity to frailty/mortality. [file JCSM-16-e13729-s001.docx]

Supplemental table 1. Association between tertiles of protein intake and mortality in individuals with multimorbidity. (N=1868)

|  | Protein intake, % energy | | | |
| --- | --- | --- | --- | --- |
|  | Tertile 1 | Tertile 2 | Tertile 3 | P for trend |
| Participants, n | 623 | 623 | 622 |  |
| **Total protein** |  |  |  |  |
| Person-years/n cases | 7303/158 | 7210/169 | 7285/163 |  |
| Model 1 | 1.0 | 1.03 (0.82, 1.28) | 1.04 (0.83, 1.29) | 0.76 |
| Model 2 | 1.0 | 1.06 (0.84, 1.33) | 1.06 (0.83, 1.34) | 0.66 |
| **Plant protein** |  |  |  |  |
| Person-years/n cases | 7287/167 | 7198/169 | 7313/154 |  |
| Model 1 | 1.0 | 0.95 (0.77, 1.19) | 0.84 (0.67, 1.06) | 0.14 |
| Model 2 | 1.0 | 0.90 (0.71, 1.14) | 0.77 (0.58, 1.01) | 0.06 |
| **Animal protein** |  |  |  |  |
| Person-years/n cases | 7259/167 | 7186/164 | 7354/159 |  |
| Model 1 | 1.0 | 0.94 (0.76, 1.17) | 0.90 (0.72, 1.13) | 0.37 |
| Model 2 | 1.0 | 0.99 (0.79, 1.24) | 0.91 (0.71, 1.16) | 0.41 |
| **Meat protein** |  |  |  |  |
| Person-years/n cases | 7177/179 | 7278/161 | 7343/150 |  |
| Model 1 | 1.0 | 0.87 (0.70, 1.08) | 0.93 (0.74, 1.16) | 0.57 |
| Model 2 | 1.0 | 0.87 (0.70, 1.09) | 0.94 (0.74 1.18) | 0.61 |
| **Dairy protein** |  |  |  |  |
| Person-years/n cases | 7287/153 | 7403/143 | 7108/194 |  |
| Model 1 | 1.0 | 0.80 (0.63, 1.01) | 1.07 (0.86, 1.33) | 0.33 |
| Model 2 | 1.0 | 0.83 (0.66, 1.06) | 1.08 (0.84, 1.39) | 0.38 |
| **Fish protein** |  |  |  |  |
| Person-years/n cases | 7190/179 | 7246/169 | 7362/142 |  |
| Model 1 | 1.0 | 1.01 (0.82, 1.25) | 0.84 (0.67, 1.05) | 0.10 |
| Model 2 | 1.0 | 1.08 (0.86, 1.35) | 0.88 (0.69, 1.13) | 0.24 |
| **Egg protein** |  |  |  |  |
| Person-years/n cases | 7247/168 | 7274/158 | 7276/164 |  |
| Model 1 | 1.0 | 1.08 (0.87, 1.34) | 1.05 (0.84, 1.30) | 0.72 |
| Model 2 | 1.0 | 1.10 (0.88, 1.38) | 1.07 (0.86, 1.34) | 0.59 |

METs: Metabolic Equivalent Tasks; BMI: Body mass index; MEDAs: Mediterranean Diet Adherence Screener

Estimates are hazard ratios (95% confidence interval).

Model 1: Cox regression model adjusted for age, sex, and number of chronic diseases at baseline.

Model 2: additionally adjusted for educational level (≤primary, secondary, or university), habitat (<10,000; 10,000-100,000; >100,000-500,000; >500,000), smoking status (never, former, current smoker), alcohol consumption (tertiles of g/d), physical activity (tertiles of METs-h/wk), hours of TV (tertiles of hours/d), sleeping time (tertiles of hours/d), number of drugs used, BMI (<25, 25–29.9, ≥30 kg/m^2^), energy intake (tertiles of kcal/d), % of saturated fat, % of monounsaturated fat, % of polyunsaturated fat, and diet quality (MEDAS score, tertiles).

All models include mutual adjustment for percentages of energy derived from each other source of protein (e.g., vegetable protein was adjusted for animal protein; dairy protein was adjusted for plant protein and all other types of animal protein, etc.).

|  | | | | | Protein intake, % energy | | |  | | |
| --- | --- | --- | --- | --- | --- | --- | --- | --- | --- | --- |
|  | N/n | Total protein | Plant protein | Animal protein | | Meat protein | Dairy protein | | Fish protein | Egg protein |
| 1-SD increment, % energy |  | 5.06 | 4.40 | 3.17 | | 1.78 | 1.63 | | 1.39 | 1.03 |
| Multimorbidity to frailty | 1868/196 | **0.81 (0.69, 0.97)** | 1.02 (0.84, 1.23) | **0.80 (0.67, 0.96)** | | 0.96 (0.82, 1.12) | 0.86 (0.73, 1.02) | | **0.82 (0.68, 0.99)** | 0.99 (0.85, 1.14) |
| Multimorbidity to mortality | 1868/407 | 0.97 (0.87, 1.09) | 0.88 (0.77, 1.00) | 0.96 (0.85, 1.07) | | 0.95 (0.85, 1.05) | 1.00 (0.89, 1.12) | | 0.99 (0.90, 1.10) | 1.02 (0.92, 1.12) |
| Frailty to mortality | 196/83 | **1.38 (1.06, 1.82)** | **1.30 (1.01, 1.68)** | **1.45 (1.06, 1.99)** | | 1.33 (0.98, 1.81) | 1.27 (0.94, 1.73) | | 1.05 (0.76, 1.46) | 1.29 (0.95, 1.76) |
| METs: Metabolic Equivalent Tasks; BMI: Body Mass Index; MEDAS: Mediterranean Diet Adherence Screener; eGFR: Estimated Glomerular Filtration Rate.  Estimates are hazard ratios (95% confidence interval).  Multistate models adjusted for age, sex, number of chronic diseases at baseline, educational level (≤primary, secondary, or university), habitat (<10,000; 10,000-100,000; >100,000-500,000; >500,000), smoking status (never, former, current smoker), alcohol consumption (tertiles of g/d), physical activity (tertiles of METs-h/wk), hours of TV (tertiles of hours/d), sleeping time (tertiles of hours/d), number of drugs used, BMI (<25, 25–29.9, ≥30 kg/m^2^), energy intake (tertiles of kcal/d), % of saturated fat, % of monounsaturated fat, % of polyunsaturated fat, diet quality (MEDAS score, tertiles) and eGFR (mL/min/1.73 m^2^).  All models include mutual adjustment for percentages of energy derived from each other type of protein (e.g., vegetable protein was adjusted for animal protein; dairy protein was adjusted for plant protein and all other types of animal protein, etc.). | | | | | | | | | | |

Supplemental table 2. Progressions from multimorbidity to frailty and mortality per 1-SD increment of protein intake, with additional adjustment for estimated glomerular filtration rate.

**Supplemental table 3. Progressions from multimorbidity to frailty and mortality per 1-SD increment of protein intake, with additional adjustment for dietary vitamin D.**

|  | | | | | Protein intake, % energy | | |  | | |
| --- | --- | --- | --- | --- | --- | --- | --- | --- | --- | --- |
|  | N/n | Total protein | Plant protein | Animal protein | | Meat protein | Dairy protein | | Fish protein | Egg protein |
| 1-SD increment, % energy |  | 5.06 | 4.40 | 3.17 | | 1.78 | 1.63 | | 1.39 | 1.03 |
| Multimorbidity to frailty | 1868/196 | **0.83 (0.69, 0.99)** | 1.03 (0.85, 1.24) | **0.82 (0.67, 0.98)** | | 0.96 (0.82, 1.12) | 0.87 (0.74, 1.03) | | **0.78 (0.61, 0.99)** | 0.98 (0.85, 1.14) |
| Multimorbidity to mortality | 1868/407 | 0.96 (0.85, 1.08) | **0.87 (0.76, 0.99)** | 0.95 (0.84, 1.07) | | 0.95 (0.85, 1.05) | 0.99 (0.89, 1.11) | | 0.99 (0.88, 1.11) | 1.02 (0.92, 1.12) |
| Frailty to mortality | 196/83 | **1.53 (1.14, 2.04)** | 1.27 (0.99, 1.65) | **1.55 (1.11, 2.16)** | | 1.26 (0.94, 1.70) | 1.22 (0.90, 1.65) | | 1.26 (0.79, 2.01) | 1.22 (0.90, 1.66) |
| METs: Metabolic Equivalent Tasks; BMI: Body Mass Index; MEDAS: Mediterranean Diet Adherence Screener; eGFR: Estimated Glomerular Filtration Rate.  Estimates are hazard ratios (95% confidence interval).  Multistate models adjusted for age, sex, number of chronic diseases at baseline, educational level (≤primary, secondary, or university), habitat (<10,000; 10,000-100,000; >100,000-500,000; >500,000), smoking status (never, former, current smoker), alcohol consumption (tertiles of g/d), physical activity (tertiles of METs-h/wk), hours of TV (tertiles of hours/d), sleeping time (tertiles of hours/d), number of drugs used, BMI (<25, 25–29.9, ≥30 kg/m^2^), energy intake (tertiles of kcal/d), % of saturated fat, % of monounsaturated fat, % of polyunsaturated fat, diet quality (MEDAS score, tertiles) and **dietary vitamin D intake (grams/d).**  All models include mutual adjustment for percentages of energy derived from each other type of protein (e.g., vegetable protein was adjusted for animal protein; dairy protein was adjusted for plant protein and all other types of animal protein, etc.). | | | | | | | | | | |

Supplemental figure 1. Participants’ flow chart.

Seniors-ENRICA

N=3289

Eligible

N=2916

Excluded:

-Missing multimorbidity status: n = 68

-Missing baseline frailty status: n = 203

-Prevalent frailty: n = 89

-Implausible or missing energy intake: n = 13

Excluded:

-No prevalent multimorbidity at baseline: n = 1048

Analytical sample

N = 1868

**
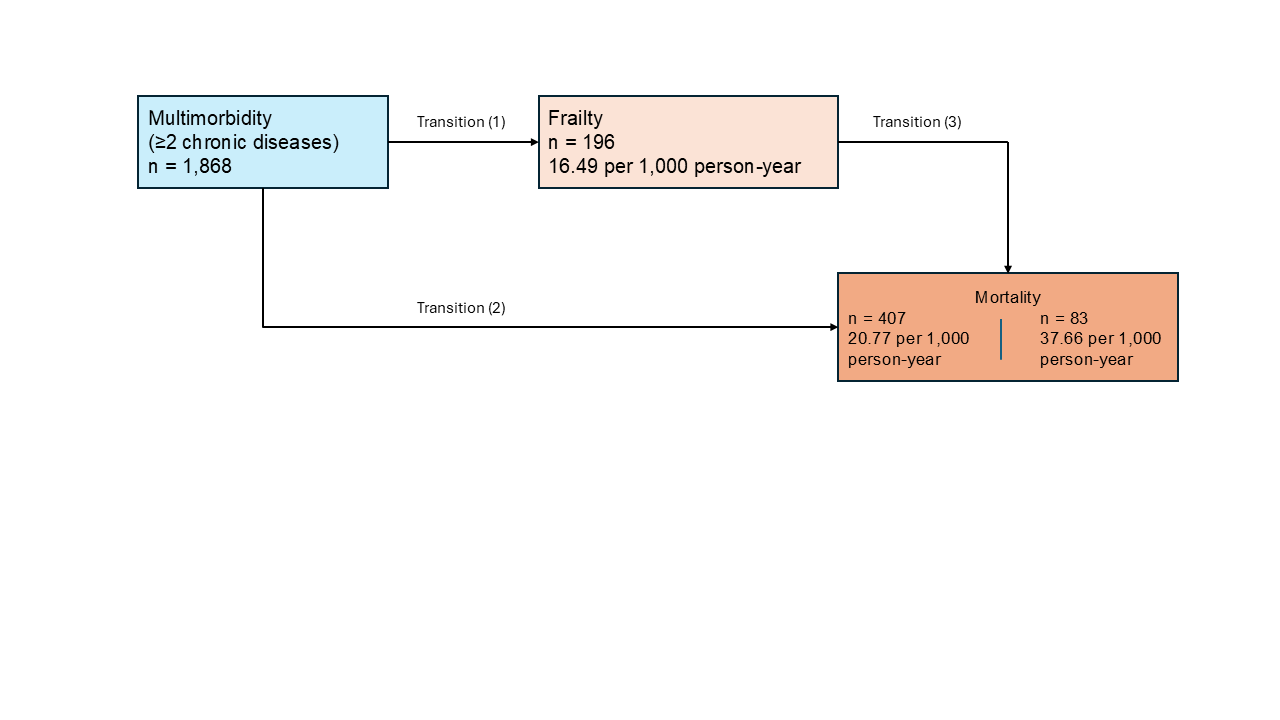
Supplemental figure 2. Progressions from multimorbidity to frailty/mortality.**

Incidence per 1000 person-years of the transitions from multimorbidity status to (1) frailty, (2) mortality or (3) frailty to mortality.

Supplementary References

[S1] Rodríguez-Artalejo F, Graciani A, Guallar-Castillón P, León-Muñoz LM, Zuluaga MC, López-García E *et al.* Rationale and methods of the study on nutrition and cardiovascular risk in Spain (ENRICA). *Rev Esp Cardiol* 2011;**64**:876–882.

[S2] Guallar-Castillón P, Sagardui-Villamor J, Balboa-Castillo T, Sala-Vila A, Astolfi MJA, Pelous MDS *et al.* Validity and Reproducibility of a Spanish Dietary History. *PLoS One* 2014;**9**:86074.

[S3] Farrán A, Zamora R, Cervera P. *Tablas de composición de alimentos del CESNID*. 2nd ed. Barcelona: McGraw-Hill/Interamericana de España SAU; 2004.

[S4] Moreiras O, Carvajal A, Cabrera L, Cuadrado C. *Tablas de composición de alimentos*. 11th ed. Ediciones Pirámide; 2007.

[S5] United States Department of Agriculture. Agricultural Research Service. USDA National Nutrient Database for Standard Reference. 2010.

[S6] Morley JE, Malmstrom TK, Miller DK. A simple frailty questionnaire (FRAIL) predicts outcomes in middle aged African Americans. *J Nutr Health Aging* 2012;**16**:601–608.

[S7] Ministerio de Sanidad - Portal Estadístico del SNS - Estadisticas y Estudio- Estadísticas Sanitarias - Índice Nacional de defunciones. Accessed 19 September 2023.

[S8] Pols MA, Peeters PHM, Ocké MC, Slimani N, Bueno-De-Mesquita HB, Collette HJA. Estimation of reproducibility and relative validity of the questions included in the EPIC Physical Activity Questionnaire. *Int J Epidemiol* 1997;**26**.

[S9] Schröder H, Fitó M, Estruch R, Martínez-González MA, Corella D, Salas-Salvadó J *et al.* A Short screener is valid for assessing mediterranean diet adherence among older spanish men and women. *Journal of Nutrition* 2011;**141**:1140–1145.

[S10] Zhang QL, Rothenbacher D. Prevalence of chronic kidney disease in population-based studies: Systematic review. *BMC Public Health* 2008;**8**:117.

[S11] Meira-Machado L, de Uña-Álvarez J, Cadarso-Suárez C, Andersen PK. Multi-state models for the analysis of time-to-event data. *Stat Methods Med Res* 2009;**18**:195–222.

[S12] Commenges D. Multi-state models in epidemiology. *Lifetime Data Anal* 1999;**5**:315–27.
